# Dataset: A proxy for historical CO_2_ emissions related to centralised electricity generation in Europe

**DOI:** 10.1016/j.dib.2021.107016

**Published:** 2021-03-29

**Authors:** Leon Joachim Schwenk-Nebbe, Marta Victoria, Gorm Bruun Andresen

**Affiliations:** a Department of Engineering, Aarhus University, Inge Lehmanns Gade 10, Aarhus 8000, Denmark; b iCLIMATE Interdisciplinary Centre for Climate Change, Aarhus University, Frederiksborgvej 399, Roskilde 4000, Denmark

**Keywords:** Carbon dioxide emissions, Energy, Electricity generation, National emission data, Historical emission values, Computed emission proxy

## Abstract

This paper presents data for the estimation of carbon dioxide (CO_2_) emissions resulting from public generation of electricity in the period from 1990 to 2018 in European countries. The base data used in the calculation of the proxy are the national emissions reported to the United Nations Framework Convention on Climate Change (UNFCCC) and the European Union (EU) Greenhouse Gas Monitoring Mechanism. Subsequently, this data is compiled and held by the European Environment Agency (EEA) from where it is accessed. The emission data is reported aggregated from thermal power stations, district heating plants, and cogeneration in combined heat and power (CHP) plants. We calculate a proxy for emissions by electricity generation alone by combining the emissions from thermal power stations and the share of CPH emissions belonging to electricity generation. The computed data was validated on the period from 2000 to 2015 by comparing it to a secondary dataset. The found emission values of the year 1990 are of particular importance as this is a commonly used emission reference year. The provided dataset, charts and figures can be reused for both analysing past emission evolutions and building models about future electricity generation emissions in Europe. The dataset is freely available in [1]. A subset of the dataset has been applied in “CO_2_ quota attribution effects on the European electricity system comprised of self-centred actors” [2] to assess the effects of potential total and national CO_2_ quota attributions in the European electricity system of the near future.

## Specifications Table

**Table:** 

Subject	Pollution
Specific subject area	Historical carbon dioxide emissions due to public electricity generation in Europe
Type of data	Table Chart Graph Figure
How data were acquired	Relying on public data entries the dataset was computed and validated using Python 3.7 running through JupyterLab 3.0.7 with the following packages: • NumPy 1.19.1 • Pandas 1.1.1 • Pyxlsb 1.0.8 • Matplotlib 3.3.4.
Data format	Data are raw, computed, analysed, filtered, and validated. Python scripts (code) as Jupyter Notebook/JupyterLab files are included.
Parameters for data collection	From the UNFCCC statistics we use the data subset ‘1.A.1.a - Main Activity Electricity and Heat Production’ and restrict the analysis to the pollutant ‘CO_2_’. The emission split is facilitated by assuming a fixed heat efficiency. We use the heat efficiency of a standard heat boiler of 90%.
Description of data collection	The dataset is based primarily on national emission statistics reported by the European countries themselves. The raw data is downloaded from the respective public sources and archived sets are extracted. See the script files (find an overview below) for details on how the raw data files are parsed and how relevant subsets are selected. Historic energy balances from Eurostat are used to disentangle the emission statistics. Lastly, data from the JRC Integrated Database of the European Energy System (JRC-IDEES) database is used to validate the calculated proxy on a narrower time period. The primary data and validation data are publicly available on the internet.
Data source location	The primary data sources and their accessibility are listed here. The input data consists of the following datasets: UNFCCC emission inventory from [3], national energy balance statistics from Eurostat [4], and the JRC-IDEES database from [5], described in the publication [6]. All utilised data is freely available. Accessing the validation data form the JRC requires a free EU Login account.
Data accessibility	The dataset and accompanying files are freely available and hosted on Mendeley Data [1]. Repository name: *Proxy for historical CO_2_ emissions related to centralised electricity generation in Europe* Data identification number: https://dx.doi.org/10.17632/cmngb4vdb5.3 Direct URL to data: https://data.mendeley.com/datasets/cmngb4vdb5
Related research article	L.J. Schwenk-Nebbe, M. Victoria, G.B. Andresen, M. Greiner, CO_2_ Quota Attribution Effects on the European Electricity System Comprised of Self-Centred Actors, Advances in Applied Energy, 2021, 100012, https://doi.org/10.1016/j.adapen.2021.100012

## Value of the Data

• A proxy for the CO_2_ emissions related solely to electricity generation is relevant in several contexts and without it, we cannot track the evolution of pollution from public electricity generation. For each included country we can analyse the past evolution and relate this to future projections and goals.
• The dataset is of crucial importance to researchers or industry professionals that are interested in the evolution of country-specific emissions due to electricity generation alone.
• The data can be used as a basis to compute future emission projections in the electricity sector or even to build software tools for modelling energy systems.
• Since the found emission values are validated using a secondary data set their reliability is high.
• The evolution of historic emissions is a significant factor in planning, simulating, and assessing the future energy system transition necessary to mitigate anthropogenic climate change.
• The CO_2_ emissions of 1990 are of notable importance as they are often used as reference scenarios for future emission reduction goals.

## 1 Data Description

As for the general public, also the energy industries have seen a grown emphasis on CO_2_ emissions which has increased significantly during the past decade pushing for fast reductions in many places. The year 1990 is often used as a reference year for emission statistics and our emission dataset, therefore, goes back to this year. Since the raw data does not exist, a proxy needs to be calculated which can be done based on different assumptions. We have established a clean methodology for calculating these solely electricity-generation related emissions. It is our belief that we found a good compromise between simplicity and accuracy, requiring only the assumption of a fixed-heat-efficiency. See the following section for further details on the methodology.

The dataset now made available was created aiming at the development of energy system models. Consider the tasks of assessing past electricity-generation related emissions or allocating future emission allowances for electricity generation in Europe. These tasks had not been easily accomplished and relied on making specific assumptions on splitting factors or different types of individual calculations for each study every time. With the publication of this dataset, and the data thereby being openly available, the same validated data with a single set of clearly defined assumptions can easily be applied to multiple future studies. Hence, saving both efforts and time while making different studies more comparable. Furthermore, the dataset can be utilised for other applications too. An example would be educators using the datasets for data mining and visualisation training or promoting understanding of which sectors contributed to emissions by how much during the years.

The following is a description of all provided data in the dataset. This description is organised around the folder structure of the dataset.

### 1.1 Repository root folder

The root folder contains three subfolders that encompass the full dataset, scripts to create it, figures visualising it, and scripts and figures validating the dataset.

#### 1.1.1 Dataset

The dataset folder contains the computed proxy for electricity emissions resulting solely from public electricity generation. Two files are found in this folder:

‐ elec_emissions.csv
 This comma-separated values (CSV) file contains the values of electricity generation related emissions measured in TgCO_2_/year (teragrams of CO_2_ per year) for each country. The values are given for all the EU-27 countries plus Great Britain and Norway for the temporal range of 1990–2018.
‐ elec_emission_incl_autoprod.csv
 Likewise, this file contains the same values for the same countries and period but also includes the estimated CO_2_ emission contribution from autoproducers.

#### 1.1.2 Scripts

The scripts folder contains all necessary computer code to produce both the entire dataset and all accompanying figures (including the validation outlined in the next section). The computation is split into three steps that each are given as a stand-alone Jupyter Notebook/JupyterLab file:

‐ data_1_calculation.ipynb
 This file contains the routines for reading in the raw data, selecting the relevant quantities, and computing the emission proxies. This code produces the files found in the dataset folder.
‐ data_2_visualisation.ipynb
 This code file includes steps to produce the figures visualising the CO_2_ emission development for both the European countries together and as single figures for each country.
‐ data_3_validation.ipynb
 The validation of the computed proxy is facilitated in this code file. In order to compare to the secondary dataset extensive reading of additional data files is necessary. The outcome of this script is two figures found in the validation part of the figures folder (as outlined below).

#### 1.1.3 Figures

All figures are to be found in this folder. It contains three subfolders as follows.

##### 1.1.3.1 The aggregated_view_figures folder

This folder is aimed at facilitating a fast and easily accessible overview of the created datasets. It contains three figures.

‐ emission_evolution_EU.png
 Showing the evolution of the CO_2_ emissions related to electricity generation in Europe with and without contributions from autoproducers from 1990 to 2018.
‐ emission_evolution_countries.png
 For each European country, the figure shows the evolution of CO_2_ emissions related to public electricity generation. The six largest emitters in 1990 are indicated and tracked over time.
‐ emission_incl_autoprod_evolution_countries.png
 For each European country, the figure shows the evolution of CO_2_ emissions related to public electricity generation including contributions from autoproducers. The six largest emitters in 1990 are indicated and tracked over time.
‐ emission_evolution_countries_pie_charts.png
 Pie charts visualising the contributions of the individual countries to the total European electricity generation related emissions for both the year 1990 and 2018.
‐ emission_incl_autoprod_evolution_countries_pie_charts.png
 Pie charts visualising the contributions of the individual countries to the total European electricity generation related emissions including contribution from autoproducers for both the year 1990 and 2018.

##### 1.1.3.2 The national_evolution_figures folder

For each of the countries, two figures are showing bar charts over the national electricity-generation related emission evolution with and without autoproducer contributions, respectively. In total, this folder contains 58 figures.

##### 1.1.3.3 The validation_figures folder

Two figures are contained in this folder.

‐ validation_secondary_data_emission_evolution.png
 Visualizing the CO_2_ emission contributing elements in the individual countries this figure provides an overview of how large individual sector contributions are in the respective countries. The three components of interest are coloured in orange, yellow, and red. These are the contributions from electricity, CHP, and heating plants. Note that for all countries that have a large CHP contribution the methodology laid out in this paper is of importance.
‐ validation_comparison.png
 The main figure of the data validation shows the two computed proxies for electricity-only related emission values (with and without autoproducer contributions) alongside their counterparts from the JRC-IDEES database. Note that autoproducer contributions are not split up for the latter case and include both heat and power generation.

## 2 Experimental Design, Materials and Methods

The procedure of data acquisition is separated into two steps: computing of the proposed proxy and subsequently a validation hereof.

### 2.1 Computing the proxy for pure electricity generation emissions

The emissions from public electricity and heat production are only reported aggregated. We detangle this data by calculating a proxy for the emissions related solely to electricity generation. The emission data from the UNFCCC does not include autoproducer contributions. We estimate their contributions and present the data with and without them. In this section, we lay out a methodology to compute the wanted proxy for historical emission values.

National emission statistics are reported to the UNFCCC in a variety of categories and subcategories. An overview of the reporting standards and categories can be found in the IPCC Guidelines for National Greenhouse Gas Inventories [7]. The IPCC emission categories relevant to this study are as follows:

• 1A1-Energy Industries
  ○ 1A1a-Public Electricity and Heat Production
    ■ 1A1a1-Public Electricity Generation
    ■ 1A1a2-Public Combined Heat and Power Generation (CHP)
    ■ 1A1a3-Public Heat Plants

In 1990 the European countries were only required to report the aggregated values of 1A1a and not split this data into its subcategories. Hence, these emission values need to be split into emissions from either heat or electricity generation. The process is further complicated by cogeneration in CHP facilities.

We split the emission data into the emissions from purely electricity, purely heat, and combined heat and power generation according to energy statistics from Eurostat. The latter is split under the assumption of the fixed-heat-efficiency approach into electricity and heat emission contributions. Applying the fixed-heat-efficiency approach, one first fixes the efficiency of heat generation. Afterwards, the input to heat generation is obtained, and subsequently one calculates the input to electricity generation as the residual from the total energy input. The assumed heat efficiency is set to that of a typical heat boiler at 90%.

The emission proxy for electricity generation related emission is calculated as follows:

$$emission_{elec-only}=\;emissions_{1A1a}\;*\;\left(\left(MAP\_EI-\left(GHP\_MAPCHP/0.9\right)\right)\;/\;\left(MAP\_EI\;+\;AP\_EI\right)\right)$$

While emissions from electricity generation with estimated contributions from autoproducers are calculated as follows:

$$\begin{array}{ccc} emission_{elec-only-incl-autoproducers} & = & emissions_{1A1a}*(\left(MAP\_EI-(GHP\_MAPCHP/0.9)\right)/\\ & & (MAP\_EI+AP\_EI))*(1+(AP\_EI-(AP\_DHO/0.9))/\\ & & \left(MAP\_EI-(MAP\_DHO/0.9))\right) \end{array}$$

Where we have the following Eurostat indicators:

**Table:** 

TI_EHG_MAPE_E	Transformation input-Electricity and heat generation-Main activity producer electricity
TI_EHG_MAPCHP_E	Transformation input-Electricity and heat generation-Main activity producer CHP
TI_EHG_MAPH_E	Transformation input-Electricity and heat generation-Main activity producer heat
TI_EHG_APE_E	Transformation input-Electricity and heat generation-Autoproducer electricity
TI_EHG_APCHP_E	Transformation input-Electricity and heat generation-Autoproducer CHP
TI_EHG_APH_E	Transformation input-Electricity and heat generation-Autoproducer heat
GHP_MAPCHP	Gross heat production-Main activity producer CHP
GHP_MAPH	Gross heat production-Main activity producer heat
GHP_APCHP	Gross heat production-Autoproducer CHP
GHP_APH	Gross heat production-Autoproducer heat

And used the following categories:

MAP_EI = TI_EHG_MAPE_E + TI_EHG_MAPCHP_E + TI_EHG_MAPH_E
AP_EI = TI_EHG_APE_E + TI_EHG_APCHP_E + TI_EHG_APH_E
MAP_DHO = GHP_MAPCHP + GHP_MAPH
AP_DHO = GHP_APCHP + GHP_APH

For the full detail of the calculation, the reader is referred to the documentation in the provided code files.

### 2.2 Validation of the computed proxy

**Fig. 1:**
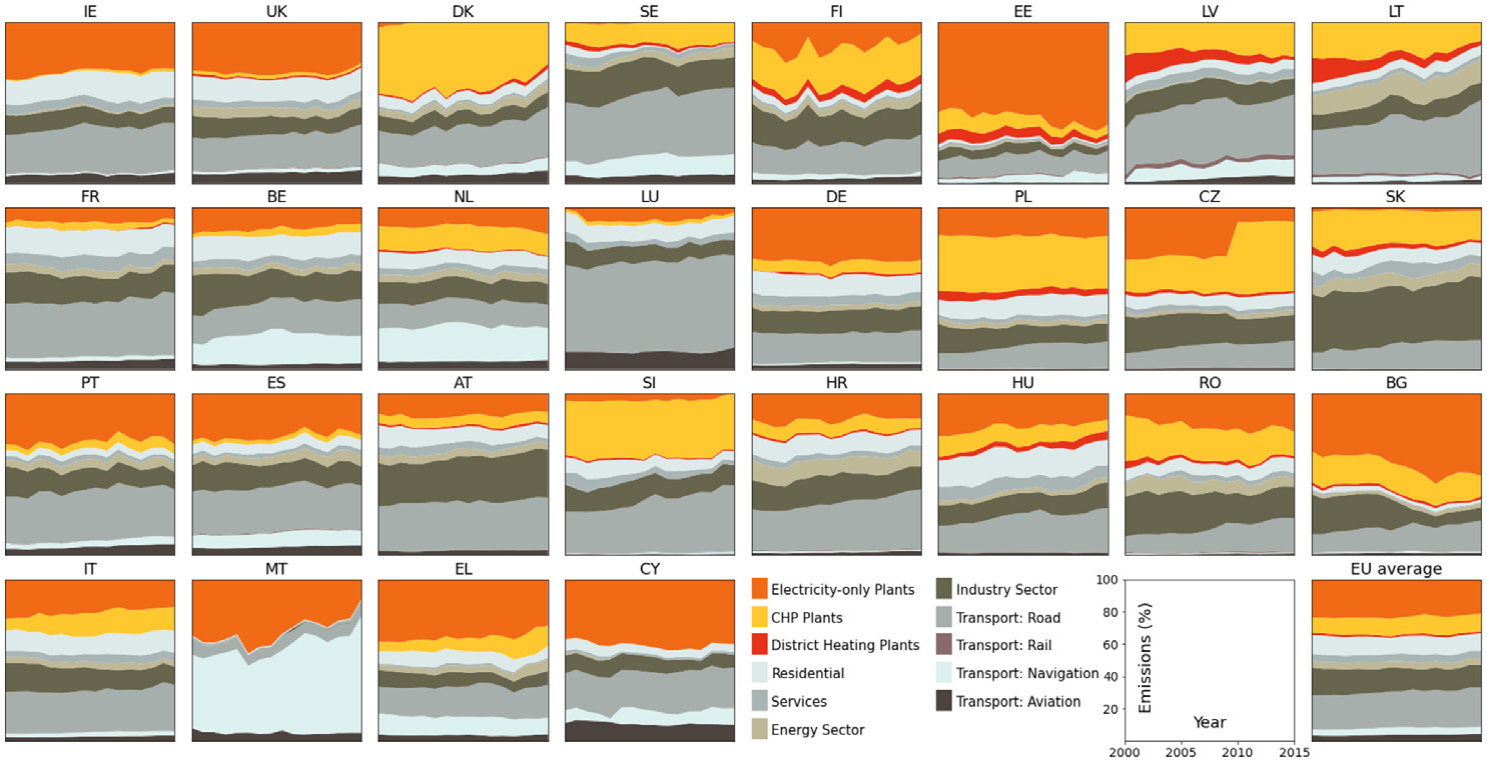
Showing the total CO_2_ emissions split into their respective contributing sectors for the EU-27 countries and Great Britain. Centralised electricity and heat production emissions are visualised in orange, yellow, and red, respectively, in the figure. The yellow parts correspond to emissions from CHP plants and need to be split into emissions related to electricity or heat production. This figure is used to show the significance of a method for splitting CHP emissions and including this contribution to electricity generation related emissions. (For interpretation of the references to color in this figure legend, the reader is referred to the web version of this article.)

Let us turn to validate the dataset. Since the calculated emission quantities are not publicly available, we cannot directly assess their validity. Instead, we compare them to another data source. The JRC-IDEES database from [5], which is described in detail in [6], offers exactly the relevant quantities but only on the timespan from 2000 to 2015. Fig. 1 visualises which sectors contribute to the national CO_2_ emissions by what degree. Countries with large CHP contributions are especially interesting as a good emission split is of larger importance here.

**Fig. 2:**
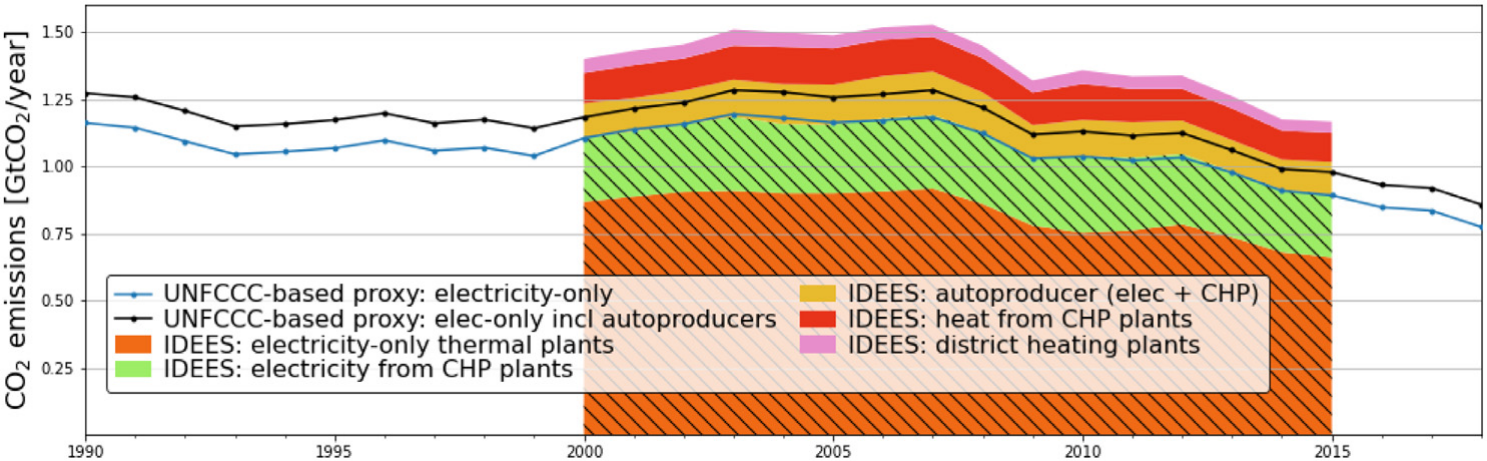
Visualising the yearly development of the part of the European emissions that are related to electricity generation from two data sources: the proposed proxy which is based on the UNFCCC data and the corresponding values provided by the JRC-IDEES database. A good agreement is observed between the two datasets. The proposed proxy comes with the benefit of a larger temporal coverage which includes the important reference year 1990. (For interpretation of the references to color in this figure legend, the reader is referred to the web version of this article.)

Fig. 2 is comparing the proposed proxy for European electricity-generation related emissions based on the UNFCCC data to the corresponding values provided by the JRC-IDEES database for the EU-27 countries together with Great Britain. The proposed proxy agrees well with the hatched area of the IDEES data representing emissions from public electricity generation and the share from CHP generation attributed to electricity generation.

### 2.3 Combining the dataset with electricity generation data

We presume that the provided data on CO_2_ emissions related solely to electricity generation will in some cases be applied alongside data on the corresponding electricity generation.

Electricity generation data can opportunely be obtained from [4]. Conveniently, the script “data_1_calculation.ipynb” found in the scripts folder with this dataset is already reading similar information, including the gross heat generation. The routine can, in straightforward manners, be adapted to read either the total power generation from each country or other associated electricity-generation quantities that might be of interest in this relation. Selected Eurostat indicators concerning this are listed in the following table:

**Table:** 

GEP_MAPE	Gross electricity generation-Main activity electricity
GEP_MAPCHP	Gross electricity generation-Main activity CHP plants
GEP_APE	Gross electricity generation-Autoproducer electricity
GEP_APCHP	Gross electricity generation-Autoproducer CHP plants

A not-far-fetched application would be the calculation of the emission intensities resulting from electricity generation. To this end, the emission values in this dataset should merely be divided by the related total power generation amounts.

## Ethics Statement

The authors declare that this work does not involve the use of human subjects or experimentation with animals.

## CRediT Author Statement

**Leon Joachim Schwenk-Nebbe:** Conceptualization, Methodology, Software, Validation, Writing - Original draft preparation; **Marta Victoria:** Supervision; **Gorm Bruun Andresen:** Supervision.

## Declaration of Competing Interest

The authors declare that they have no known competing financial interests or personal relationships which have or could be perceived to have influenced the work reported in this article.

## References

[1] Schwenk-Nebbe L. (2021). Proxy for historical CO_2_ emissions related to centralised electricity generation in Europe. Mendeley Data.

[2] Schwenk-Nebbe L.J., Victoria M., Andresen G.B., Greiner M. (2021). CO_2_ quota attribution effects on the European electricity system comprised of self-centred actors. Adv. Appl. Energy.

[3] European Environment Agency (EEA), (2020). National Emissions Reported to the UNFCCC and to the EU Greenhouse Gas Monitoring Mechanism. https://www.eea.europa.eu/ds_resolveuid/a6e1bc85fbed4989b0fd6739c443739a.

[4] Statistical Office of the European Union (Eurostat) (2020). Energy balances, Energy Balances in the MS Excel file Format (2020 Edition). https://ec.europa.eu/eurostat/web/energy/data/energy-balances.

[5] L. Mantzos, N.A. Matei, E. Mulholland, M. Rózsai, M. Tamba, T. Wiesenthal, JRC-IDEES 2015. European commission, Joint Research Centre (JRC), v1, 2018. http://data.europa.eu/89h/jrc-10110-10001. Accessed February 1, 2021.

[6] Mantzos L., Wiesenthal T., Matei N.A., Tchung-Ming S., Rozsai M., Russ P., Soria Ramirez A. (2017). JRC-IDEES.

[7] Sanchez M.J.S., Bhattacharya S., Mareckova K. (2006). 2006 IPCC Guidelines for National Greenhouse Gas Inventories. https://www.ipcc-nggip.iges.or.jp/public/2006gl/pdf/1_Volume1/V1_8_Ch8_Reporting_Guidance.pdf.

